# Editorial: Sex Differences in the Autistic Brain

**DOI:** 10.3389/fnint.2021.708368

**Published:** 2021-06-15

**Authors:** B. Blair Braden, Gregory L. Wallace, Dorothea L. Floris, Kevin A. Pelphrey

**Affiliations:** ^1^College of Health Solutions, Arizona State University, Tempe, AZ, United States; ^2^Department of Speech, Language, and Hearing Sciences, The George Washington University, Washington, DC, United States; ^3^Donders Centre for Cognitive Neuroimaging, Donders Institute for Brain, Cognition and Behaviour, Radboud University, Nijmegen, Netherlands; ^4^Department of Neurology, University of Virginia, Charlottesville, VA, United States

**Keywords:** autism, sex differences, neuroscience, cognition, neuroimaging

Diagnostic characterizations of autism spectrum disorder (ASD) have largely been based on the male phenotype. Thus, diagnostic assessments of core ASD symptoms show poorer sensitivity in females than males (Lai et al., 2011), resulting in missed or delayed identification and reduced availability of support services for females with ASD. Emerging research shows ASD-related behaviors are qualitatively different in females (Werling and Geschwind, 2013), but detecting these differences is variable across the lifespan (Reinhardt et al., 2015).

Research into the neurobiological underpinnings of such sex differences is still in its infancy, but understanding the brain-basis of sex differences in ASD will be paramount to accurately diagnosing females and providing optimal sex-specific interventions. Emerging research finds persistent brain differences in older adults with ASD, compared to neurotypical (Koolschijn et al., 2017; Walsh et al., 2019), yet sex differences in this age group are under-explored. Further, brain-based biomarkers are a goal for precision medicine in ASD but have proven elusive because of heterogeneous factors, such as sex differences (Uddin et al., 2017).

The goal of this Research Topic was to build knowledge on sex differences in the autistic brain in order to lay the groundwork for sex-specific, neurobiologically-informed diagnostics and interventions. Authors were encouraged to organize their work around the themes of neural sex differences across the lifespan, neural correlates of behavioral sex differences, and sex-specific brain-based biomarkers or interventions in ASD.

First, Kirkovski et al. extended previous work using cutting-edge diffusion-weighted imaging metrics in adolescents and young-adults (Dimond et al., 2019) to an older group of adults with ASD. Thus, they represent only the second group to use these innovative metrics that can account for crossing fibers and provide indices of white matter micro- and macro-structure in ASD. Dimond et al. (2019) application in found reduced fiber density within the corpus callosum, inferior fronto-occipital fasciculus bilaterally, as well as the right arcuate and uncinate fasciculi in adolescents and young adults with ASD, compared to neurotypicals. Very few females were included in this study, leaving unanswered questions about sex differences. In a broad age range adult sample, Kirkovski et al. found only females with ASD had reduced fiber density and cross-section, a combined metric comprised of micro- and macro-structural measures, in the corpus callosum, a difference not detected between the male ASD and neurotypical groups. From a developmental perspective, this might suggest that fiber density atypicalities normalize with age in males with ASD, but not females with ASD which should be examined in longitudinal cohorts. These findings have implications for sex-specific brain-based biomarkers in ASD that could differ across the lifespan.

Pagni et al. also addressed sex differences in lifespan development by investigating the influence of age and sex on social cognition via the Reading the Mind in the Eyes test. Social cognition declines non-linearly with age, and while sex differences have been detected in neurotypical adults (O'Brien et al., 2013; Baron-Cohen et al., 2015), they are not found in adults with ASD (Baron-Cohen et al., 2015). However, the influence of aging on social cognition in ASD had been largely ignored. In a sample of 95 adults with ASD and 82 NT adults ages 18–71 years, Pagni et al. replicated previous findings of reduced social cognition abilities in adults with ASD, compared to neurotypical adults but age and sex interactions were non-significant. It is noted that visual inspection of quadratic age curves indicated the possibility of unique social cognitive trajectories in men and women with and without ASD that should be investigated in larger longitudinal studies. Furthermore, investigating the neural correlates of these potentially age-dependent behavioral sex differences in ASD is warranted.

Prosperi et al. filled a gap in understanding sex differences on core symptoms and psychiatric comorbidities in preschoolers with ASD. This topic has received considerable attention in older child, adolescent, and adult groups, but not preschoolers. Further, the work that does exist specific to preschoolers includes few female participants and heterogeneous intellectual abilities (Little et al., 2017). In a preschool sample of 107 ASD females without non-verbal intellectual impairment who were matched one to one with 107 males, Prosperi et al. found ASD females did not differ from ASD males on core symptom severity and showed lower levels of emotionally reactivity, anxious-depression, internalizing problems, and DSM-Oriented Scales of anxiety problems. Findings support the developmental lifespan theory that core symptom sex differences may emerge in later childhood which has clear implications for optimizing ASD behavioral diagnostics and interventions.

Finally, Aykan et al. also addressed sex differences in a core ASD symptom, atypical sensory experiences. Sex differences in sensory processing in the neurotypical population are well-established, but largely unexplored in ASD. To begin addressing this gap, the authors examined the influence of autistic traits on neural auditory and visual processing in neurotypical men and women. Aykan et al. found auditory sensitivity scores for males and visual sensitivity scores for females correlated with autistic traits. Moreover, an altered auditory processing electroencephalography signature correlated with greater autistic traits only in males. Findings emphasize the importance of considering sex differences in sensory processing and should be investigated in clinical ASD cohorts across the lifespan. If validated in ASD, auditory EEG signatures may be a neural correlate and biomarker for sensory sensitivity sex differences.

In conclusion, this Research Topic makes significant contributions to understanding sex differences in ASD across the lifespan by examining white matter characteristics, social cognition, core symptoms, and psychiatric co-morbidities in some of the youngest and oldest individuals with ASD. Advanced MRI diffusion metrics and auditory EEG signatures emerge as potential sex-specific brain-based biomarkers. These are important steps for understanding sex-related heterogeneity in ASD and moving toward precision medicine.

## Author Contributions

BB led the editorial preparation. GW, DF, and KP provided critical feedback. All authors contributed to the article and approved the submitted version.

## Conflict of Interest

The authors declare that the research was conducted in the absence of any commercial or financial relationships that could be construed as a potential conflict of interest.
